# Self-shaping liquid crystal droplets by balancing bulk elasticity and interfacial tension

**DOI:** 10.1073/pnas.2011174118

**Published:** 2021-03-31

**Authors:** Karthik Peddireddy, Simon Čopar, Khoa V. Le, Igor Muševič, Christian Bahr, Venkata. S. R. Jampani

**Affiliations:** ^a^Dynamics of Complex Fluids, Max Planck Institute for Dynamics and Self-Organization, 37077 Göttingen, Germany;; ^b^Department of Physics and Biophysics, University of San Diego, San Diego, CA 92110;; ^c^Faculty of Mathematics and Physics, University of Ljubljana, 1000 Ljubljana, Slovenia;; ^d^Department of Chemistry, Faculty of Science, Tokyo University of Science, 162-8601 Tokyo, Japan;; ^e^Condensed Matter Physics Department, Jožef Stefan Institute, 1000 Ljubljana, Slovenia;; ^f^Physics and Materials Research Unit, University of Luxembourg, L-1511 Luxembourg City, Luxembourg

**Keywords:** anisotropic liquids, liquid crystals, shape transformation, vesicles, interfacial tension

## Abstract

The shape diversity and controlled reconfigurability of closed surfaces and filamentous structures, universally found in cellular colonies and living tissues, are challenging to reproduce. Here, we demonstrate a method for the self-shaping of liquid crystal (LC) droplets into anisotropic and three-dimensional superstructures, such as LC fibers, LC helices, and differently shaped LC vesicles. The method is based on two surfactants: one dissolved in the LC dispersed phase and the other in the aqueous continuous phase. We use thermal stimuli to tune the bulk LC elasticity and interfacial energy, thereby transforming an emulsion of polydispersed, spherical nematic droplets into numerous, uniform-diameter fibers with multiple branches and vice versa. Furthermore, when the nematic LC is cooled to the smectic-A LC phase, we produce monodispersed microdroplets with a tunable diameter dictated by the cooling rate. Utilizing this temperature-controlled self-shaping of LCs, we demonstrate life-like smectic LC vesicle structures analogous to the biomembranes in living systems. Our experimental findings are supported by a theoretical model of equilibrium interface shapes. The shape transformation is induced by negative interfacial energy, which promotes a spontaneous increase of the interfacial area at a fixed LC volume. The method was successfully applied to many different LC materials and phases, demonstrating a universal mechanism for shape transformation in complex fluids.

## Significance

Liquid crystal (LC) research is rapidly expanding to include studies of curved and topologically nontrivial structures. Here, we explore the role of the bulk LC elasticity and interfacial free energy under weak thermal stimuli to achieve structural transformations in LC emulsions using two different surfactants. Our method is universal and could be used for any LC material or phase. A theoretical model for transforming LC emulsions into uniform fibers and vice versa is presented. We also show the self-shaping of smectic vesicle structures and monodispersed droplet formation at the nematic–smectic transition, utilizing the LC bulk elasticity. This work shows the potential to obtain the controllable shape of complex curved structures for a constant volume of different LCs and other soft materials.

Interfacial tension plays a central role in the phase separation of binary mixtures into superstructures, such as droplet emulsions, filaments, and more complex objects. It is of profound importance for an understanding of structure formation on all the length scales of complex fluids, which is crucial for the mechanical characteristics, light–matter interactions, and the advancement of the physical and chemical properties of liquids (1). The manipulation of the spherical shape of a droplet of an isotropic liquid into an elongated filament or other complex shapes is possible with the use of external fields (2), cooling (3), spontaneous emulsification (4, 5), interfacial freezing (6), and dissolution (7). Greater shape variability can be achieved in systems with complex curvature-dependent membrane elasticity, which is exploited well in intracellular biological matter, vesicles, and organelles (8–11). The interfacial effects of the membrane complement the active dynamics of the cytoskeleton to produce the characteristic shapes of different types of cells, as well as self-propulsion (12), self-division (13), chemotaxis (14), and active mass transport across the membrane, which are the key attributes of a living cell (15). In anisotropic materials, most notably liquid crystals (LCs) (16), interfacial effects are employed for the formation of droplets, shells, and topological defect structures with complex properties derived from bulk elasticity and topology (17–20), which can be used for the design of smart materials that respond to selected external stimuli (21).

The self-organizing nature of anisotropic LC mesogens results in birefringent materials with long-range orientational order in the nematic (N) phase and an additional one-dimensional positional order in the smectic-A (SmA) phase (16). The average direction of an LC’s molecular alignment, defined as the director ***n***, acts as an optical axis, making LCs suitable for various photonic applications (22–25). Variation of the orientational order in the bulk gives rise to a change in the elasticity, described by the elastic constants pertaining to the splay (*K*_11_), twist (*K*_22_), and bend (*K*_33_) deformation modes. Controlled director deformation with external fields gives the extra advantage of the wide tunability of LC droplets’ optical properties that give them the potential to serve as soft photonic elements [i.e., waveguides, microresonators, and microlasers (26–28)] for which precise control over the size and shape is crucial. For example, smectic fibers grown in aqueous surfactant solutions prove to be excellent light guides for photonic applications (29).

Over two decades ago, spontaneous filament formation was reported in a number of partially ordered liquids, specifically in binary mixtures of thermotropic LCs, which form the SmA phase while cooling from the isotropic phase or through the coexistence region of two phases (30–34). In these cases, the volume of the filaments is not conserved, which contrasts with our experiments, in which the volume of the LC is conserved and only a shape transformation takes place. Later, specially designed amphitropic surfactants were shown to transform a droplet of LC into an LC fiber during fast cooling to below the Krafft temperature of the surfactant (35). The N filaments are, in this case, transient and unstable structures, which collapse back to droplets at equilibrium. Recently, it was reported that LC polydisperse oligomers, dispersed in water containing a surfactant, form dendritic N filaments (36) and specially designed fluorinated LCs form smectic filaments (37) while cooling. These reported systems require increasingly advanced approaches and special LC materials to control the growth of the filamentous structures and to bypass the shortcomings in the control, stability, and reversibility of filamentary structures, which are in most cases structurally unstable or appear only in a narrow temperature range.

In this study, we develop and systematically analyze a simple method for the creation and reversible transformation of multiple N and smectic superstructures in an aqueous host liquid. The key novelty of our method is the use of two different surfactants: one is dissolved in a droplet of LC and the other is dissolved in the water surrounding the LC droplet. The method is generic and works with very different, readily available thermotropic LCs and surfactants, which allows us a free choice of material for a desired application. We explore the interplay between the orientational elasticity of LCs and the surfactant-affected interfacial tension that leads to the shape transformation of a spherical LC droplet into single and branched fibers of uniform thickness. In addition, we form monodisperse droplets via the jet instability of the fiber driven by elastic constants of the LC and the change of the topological constraints across the phase transition into the SmA phase. Finally, we show how different life-like vesicles are spontaneously self-shaped from a single droplet in the SmA* phase and the tranformation of non-chiral SmC droplet into a helical fiber.

## Results

### Self-Shaping in the N Phase.

**Fig. 1.**
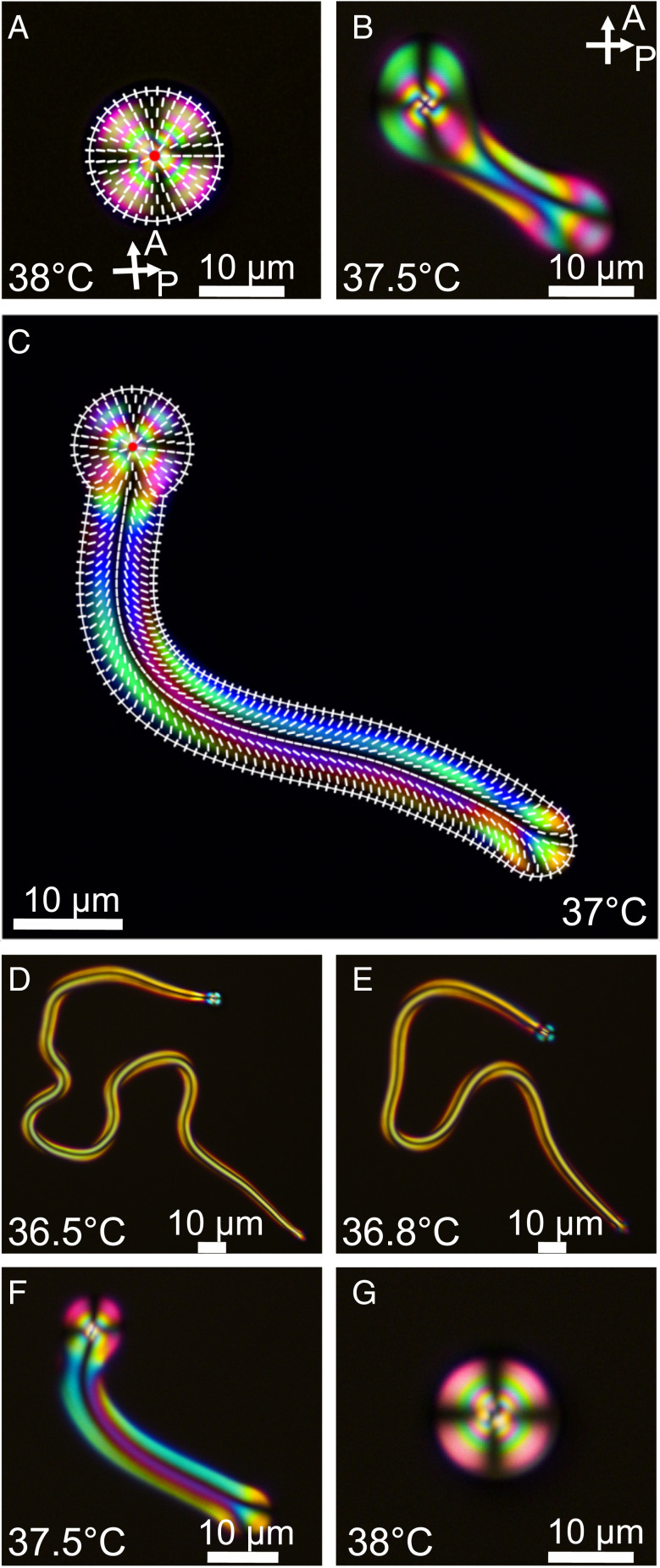
A reversible, self-shaping N LC droplet under controlled temperatures. (*A*) A snapshot of an 8CB LC droplet in the N phase with a radial director field taken between slightly uncrossed polarizers. The superposed image shows the radial director profile. (*B*) The onset of the shape transformation of the spherical droplet to a uniformly thick fiber. (*C*) A droplet with an ER fiber annotated with the director field. The white outline uses the theoretically predicted fiber-to-droplet diameter ratio, showing a good agreement with the experiments. (*D*–*G*) The shape recovery of a long ER fiber to the radial droplet after heating to the starting temperature (Movie S1 and *SI Appendix*, Fig. S3). It is worth noting that the self-shaped N fiber is stable without any observable change for at least for 10 min at any given temperature.

The primary LC material used in our experiments (8CB; Cr 21.1 °C SmA 33 °C N 40.5 °C I) is doped with a small amount of a nonionic surfactant called monoolein (dispersed phase). An aqueous solution of a cationic surfactant (CTAB) is chosen as the continuous phase for our experiment (*SI Appendix*, Fig. S1). We should stress that the use of two different surfactants is crucial for all the observed shape transformations; if only a single surfactant is used, no shape transformation is observed in any of the cases studied. A droplet of LC suspended in a CTAB surfactant solution exhibits a radial director configuration, as shown in Fig. 1*A*. With decreasing temperature, from 39 to 36 °C, still in the N phase, the spherical droplet becomes unstable, and a uniformly thick fiber starts budding and extending from it, with the director spanning continuously from the axial orientation at the center line to matching the perpendicular boundary condition at the surface (an escaped radial [ER] director), as evidenced by the polarized optical micrographs (Fig. 1 *B*–*D*). This transformation is reversible, and a spherical droplet is recovered upon reheating (Fig. 1 *E*–*G*). The fiber diameter decreases with lower temperature (Movie S1). In our experiments, the initial volume of the droplet is conserved, whereas previously reported binary mixtures of smectic LC compounds in refs. 30–34 and 37 show no volume conservation due to either partial or completely miscible LC mixtures in the continuous phase.

**Fig. 2.**
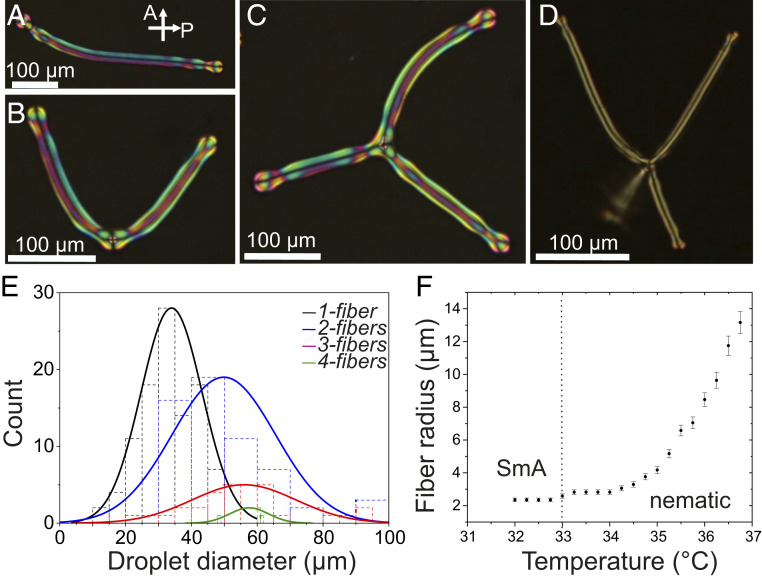
Single- and multiple-fiber structures from a single radial N droplet. (*A*) A snapshot of single fiber’s formation from the radial droplet. The initial droplet with a point defect is on the top left. (*B*) A snapshot of two fibers from a single radial droplet at the bottom of the image. (*C*) Three fibers distributed at about a 120° angle in the focal plane from a radial droplet in the center. (*D*) Four identical fibers from a single droplet in the center. One of the fibers is out of the focal plane, forming a tetrahedral arrangement. Note that each snapshot in *A*–*D* is taken from a different experiment with a different initial droplet diameter and temperature. (*E*) Normal distribution of the number of fibers versus the initial droplet diameter. (*F*) The diameter of the growing ER fiber taken at several intervals until the fiber instability takes place at the transition. The fiber diameter gradually reduces while cooling to the SmA transition.

The outcome of the fiber formation depends on the size of the droplet. Small droplets start with a spherical shape having a radial director structure and grow up to four fibers, depending on the initial diameter of the droplet. Repeated experiments of fiber formation show a high probability of forming a single fiber from an initial droplet diameter in the range 10 to 50 µm (Fig. 2 and *SI Appendix*, Fig. S2). The formation of two or three fibers occurs in droplets of more than 30 to 100 µm with four fibers only rarely being observed. The number and initial positions of the newly forming fibers are nondeterministic, initiated by small symmetry-breaking surface undulations. The probabilities of forming different numbers of fibers depend on the initial droplet diameter, as shown in Fig. 2*E* and *SI Appendix*, Figs. S2–S5. The fiber footholds repel, forming symmetric arrangements around the central droplet containing a single point defect. In addition to the fibers extending directly from the central droplet, additional branching can occur further down the fiber, creating complex dendrite structures (*SI Appendix*, Fig. S4). Such a robust control of the droplet-to-fiber transformation, with a correlation between the droplet diameter and the number of fibers, is a significant improvement beyond state of the art. Previously reported systems by Toquer et al. (35) demonstrated only the transient formation of a single fiber using rapid cooling, and Wei et al. (36) reported the dendritic growth of branched fibers using polydisperse oligomers, without control over the number of fibers. The control over the number of fibers is, in our case, not possible for much larger droplets (initial diameter >200 µm), which have an irregular initial shape with no apparent central radial defect structure. However, the fibers that grow from such large droplets all have identical thickness (Movie S2 and *SI Appendix*, Fig. S5).

### N–SmA Phase Transition-Driven Fiber Instability.

**Fig. 3.**
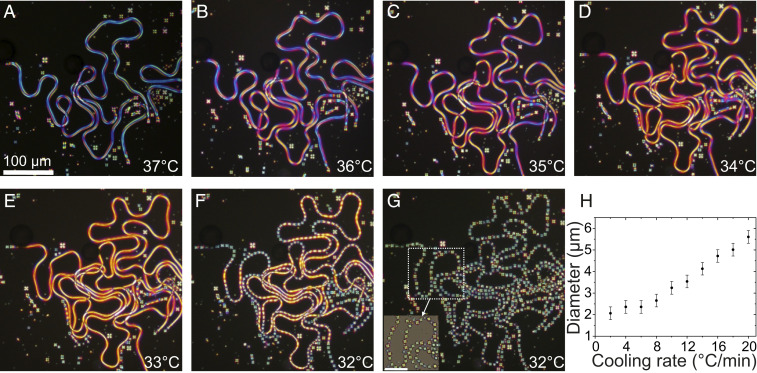
Monodisperse droplet generation while cooling to *T*_*N*–SmA_ phase transition. (*A*) A snapshot of a long single ER fiber’s formation while cooling at a rate of 1 °C/min. (*B*–*E*) Thinning of the fiber while cooling. The distinct color in each frame indicates the reduced birefringence due to the decreasing thickness. (*F*) The onset of fiber instability at the N–SmA transition. (*G*) An irreversible change of uniform droplets of size ranging from 9.9 to 10.8 µm formation at N–SmA transition, and the inset image shows the similar birefringent color of the droplets arranged in the path of the fiber (*SI Appendix*, Fig. S6). (Scale bar, 50 µm.) (*H*) The tunable diameter of SmA droplets after fiber instability at different cooling rates (Movie S3 and *SI Appendix*, Fig. S4).

The fiber elongation continues (fixed volume of LC) during cooling until the phase transition of N to SmA. The layered structure of the SmA is incompatible with the bend deformation of the escaped radial configuration due to the increase and divergence of the elastic constants ratio *K*_33_/*K*_11_ while approaching, and at, the *N*–SmA transition (38–40). In a smectic fiber, the ER director can either realign radially into concentric layers around a fiber core (*SI Appendix*, Fig. S7) or break into droplets (*SI Appendix*, Fig. S6), with the latter being more energetically favorable (*SI Appendix*). Our experiments show that spontaneous elongation and thinning of the fiber is suppressed while approaching the SmA transition (Fig. 2*F*) and eventually breaks into droplets at the *N*–SmA transition (Fig. 3 and Movie S3). This observation is somewhat similar to the report of Lavrentovich and Nastishin (41), which shows that the cholesteric-to-SmA transition leads to a division of droplets. When the elastic constants ratio exceeds 1, the core region becomes increasingly deformed, and a Rayleigh-like pearling instability of the fiber is triggered, similar in behavior to the instability seen during sudden heating by Toquer et al. (35, 41) and reminiscent of the pearling behavior of lipid bilayer membranes (11, 42). However, in contrast to the traditional Rayleigh instability, this process involves diverging the elastic energy cost related to the director bend present in the ER texture established in the N phase (41). With a faster cooling rate, the transition is reached while the fibers are still growing, which results in thicker fibers, and subsequently a larger diameter of the monodisperse droplets. Thus, the self-shaping process of a droplet-to-fiber formation and the instability triggered during the *N*–SmA transition offer precise tuning of the diameter of the droplets by varying the cooling rate (Fig. 3*H* and Movie S4). Note that the self-shaping is not limited to any one particular LC compound (*SI Appendix*, Fig. S8). A large number of different N LCs have been tested in the experiments, with the same combination of monoolein and CTAB surfactants, always resulting in the growth of N fibers from spherical N droplets.

### Self-Shaping in Smectics.

**Fig. 4.**
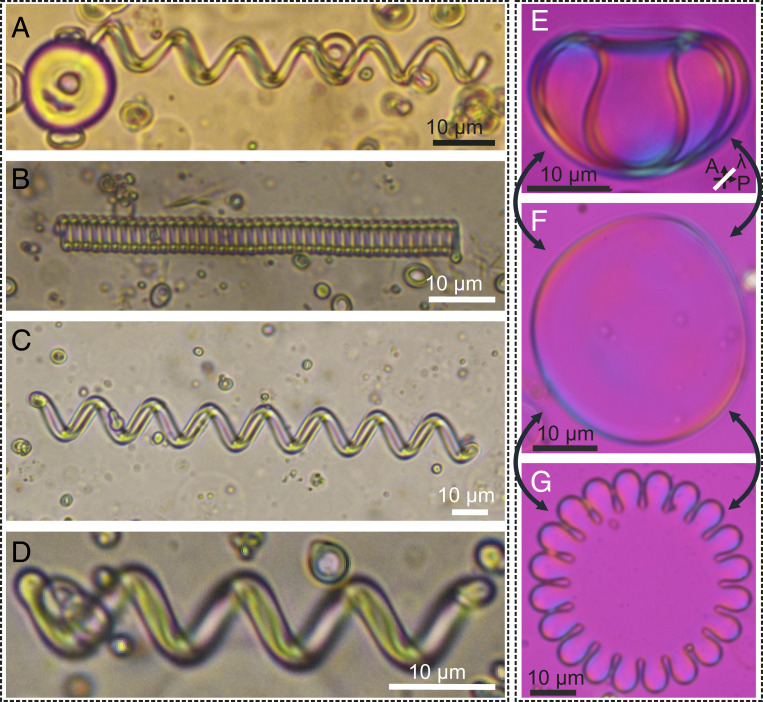
Self-shaping phenomenon in chiral smectic compounds. (*A–D*) Demonstration of self-shaping in SmC compound. (*A*) Left-handed chiral fiber growing from a SmC droplet. (*B*–*D*) Fully transformed, tightly packed, left-handed fiber and loosely packed left-handed and right-handed fibers, respectively. The experiment is carried out with 1 wt% monoolein at 1.5 mM CTAB solution at 40 °C. (*E*–*G*) Self-shaping vesicle structures in the SmA* phase. Stomatocyte shape (*E*) and starfish shape (*G*) formed from an initial discoid shape, an example of which is shown in F. (*E*–*G*) Images are taken between crossed polarizers with a waveplate (530 nm, *l*: slow axis) inserted in the optical path. The yellow and blue interference colors at the edges indicate that the smectic layers are along the edges. More detailed shape transformations of vesicles—self-closed smectic LC film containing aqueous CTAB solution on both sides—are provided in *SI Appendix*, Fig. S9.

The balance between the elastic contributions that are characteristic of each LC phase and the surfactant’s interfacial effects can be exploited in different ways, depending on the initial conditions during the self-shaping. The reversible self-shaping of droplets to fibers and vice versa is not only limited to various N LCs but is also observed for chiral and nonchiral smectics. Fig. 4 *A*–*D* shows smectic helices, which were self-transformed from a droplet of nonchiral smectic-C (SmC) material with added monoolein, floating in water with CTAB. First, a droplet of SmC material is formed in the water at a temperature at which the SmC droplet is in the isotropic phase. After the droplet is cooled to the SmC phase, smectic fibers start growing from the droplet (Fig. 4*A*) until the whole droplet is transformed into a single helical fiber. Left- and right-handed helices are equally probable, and there is always a single fiber grown from a single droplet; see Fig. 4 *B*–*D*. The helical period varies from fiber to fiber.

If the cosurfactant (monoolein) is added to droplets of the chiral smectic LC C7 and the droplets are dispersed in an aqueous CTAB solution with a concentration slightly above the critical micelle concentration (CMC), each droplet spontaneously transforms to many vesicle structures when the temperature is decreased from the isotropic to the SmA* phase of C7; see Fig. 4*F*. These vesicle structures are actually in the form of LC shells of different sizes (i.e., of a few tens of micrometers) and thicknesses (>1 µm) in the SmA* phase similar to the report of Lopez-Leon and Fernandes-Nieves (18), consisting of a self-closed smectic LC film enclosed by an aqueous CTAB solution as the inner core and suspended in the same inner aqueous CTAB solution as a continuous outer medium (*SI Appendix*, Fig. S9). Surprisingly, these smectic vesicles show a plethora of spontaneous self-shaping transformations into spontaneously curved vesicles upon cooling by a few degrees in the same phase, as shown in Fig. 4 *E* and *G*. This phenomenon makes possible the decreasing trend of interfacial tension coupled to the elastic constants for transforming into spectacular birefringent structures analogous to biomembranes, such as stomatocyte (Fig. 4*E*) and starfish shapes (43) (Fig. 4*G*). This vesicle formation is observed in a number of chiral or nonchiral smectic phases of different LCs, as long as the two surfactants are used: one in the LC and the other in water. The striking feature of vivid shape change without any sharp kinks within a few degrees, change in temperature is attributed to avoiding the bend deformation (*SI Appendix*, Fig. S10). Therefore, it is safe to convey that the tunable curvature in the LC vesicles is a result of the tendency for increasing the surface area, while simultaneously adapting the shape to reduce the elastic energy. Any detailed investigation of the elastic energy owing to a continuous shape transformation upon cooling is well beyond the scope of the current study. Nevertheless, the experimental demonstration clearly shows that the thermotropic smectic phase forming LCs can be formed into shapes that have long been known and observed in lyotropic systems (44, 45), while retaining all the benefits of thermotropic LC, such as birefringence and an electric field response.

## Discussion

At a fixed volume, a thicker, shorter N fiber has both a lower free energy due to a lower elastic deformation as well as a smaller interfacial area compared to a thinner and longer fiber. Thus, in the conventional case of a positive and isotropic interfacial energy, any N fiber would retract back into a spherical N droplet that minimizes the surface area and the elastic deformation. A gradual increase in the surface area for a given volume of a self-shaping N droplet indicates that the interfacial energy is reduced during the surface growth. To counter the increasing bulk elastic energy of the growing fiber and allow the fiber radius to stabilize, the surface-energy contribution must be negative. A similar note on negative surface energy was reported by Lavrentovich and Nastishin (41) for a sphere–fiber transformation in a narrow temperature interval near the cholesteric–SmA transition. This scenario is associated with a spontaneous growth of the surface area observed in oil droplets with tunable interfacial tension at equilibrium (sphere-to-icosahedron at positive interfacial tension) and out-of-equilibrium (icosahedron-to-fiber [or platelet] transition at negative interfacial tension) (6), while, in our case, the droplet-to-N fiber transformation takes place through near-equilibrium temperature changes. In our case, the bulk N elasticity can counter the surface growth so that a long-term, stable, and semi-equilibrium state can be reached, with no apparent macroscopic dynamics. Our structures are stable for a few tens of minutes at any given temperature (further stability can be achieved with an increased viscosity, *SI Appendix*, Fig. S7). A similar balance of positive and negative surface-scaling energy terms was also suggested to stabilize the self-shaping of oil droplets (3, 46), albeit this mechanism does not seem to be supported by some of the recent experimental evidence (47, 48). Note that this phenomenological inference does not reveal the microscopic origin of such behavior, which encompasses the effects of both surfactants and their distributions in the dispersed (LC) and continuous (water) phase, respectively. It is very indicative that self-shaping takes place only when two different surfactants are used simultaneously: one in the dispersed and the other in the continuous phase. For example, C12E3/SDS surfactant combination can be used instead monoolein/CTAB in the dispersed and continuous phases, respectively. This leads to the conjecture that both surfactants meet at the interface and in some way induce the spontaneous growth in the area of the interface. This interface acts as a thin membrane, enclosing a fixed volume of the LC. If the surface area of this membrane changes, the shape of the enclosed LC volume also has to change. A detailed investigation of this phenomenon warrants further specialized studies.

Unlike in the smectic phase, in which the mechanics of fiber growth is rather complex [described in detail by Palffy-Muhoray (49); see also refs. 33 and 50], the fiber formation in N LCs is a result of the balance between the surface- and bulk-energy contributions. Therefore, N fiber growth allows us to draw simple qualitative relations between the equilibrium fiber state and the material parameters. The bulk contributions are due to the elastic deformation (K ∼5 pN for 8CB at 35 °C). A phenomenological calculation of this balance yields a prediction of the fiber radius $r_{f}=-\frac{\text{Ω}K_{11}}{\gamma_{eff}},$ where $\gamma_{eff}$ is the effective interfacial tension and Ω≈3 slightly depends on the elastic constants, in particular on the ratio of the bend and splay elastic constant $\beta =\frac{K_{33}}{K_{11}}\approx 1$. The model also gives the droplet-to-fiber diameter ratio $\frac{r_{d}}{r_{f}}\approx 1.6$ (details are given in *SI Appendix*), which indeed matches the experimental observations well (one example is shown in Fig. 1*C*). At equilibrium, the fiber thickness and the radius of the central droplet that contains the main point defect are both independent of the initial volume of the droplet, which only reflects in the length of the fiber and the number of fibers and branches, as shown in Fig. 2. The ratio of the radii does not depend on the interfacial tension and relates directly to the elastic constant anisotropy. The approximation of a perfectly spherical droplet is relatively good for a single ER fiber, but for 2 to 4 ER fibers, the approximation becomes progressively worse, as most of the droplet surface is occupied by the fibers, and the internal director is not perfectly radial anymore.

This analysis shows that N fibers are only stable if the effective interfacial tension is negative. Thus, an increase in the surface area is energetically favorable, as discussed in ref. 41 (*SI Appendix*, Fig. S10*E*). Note that the effective interfacial tension encompasses all contributions to the free energy that scales with the surface area, not simply the isotropic surface tension we know from regular liquids. This includes the surface tension of both aqueous and LC mixtures and the mixing and orientational free energy of the surfactant–cosurfactant layer at the interface, with anisotropic dependence due to the surface anchoring (for a similar discussion, see ref. 41). Despite the preference for increasing the surface area, the fibers are stable in the N phase due to the bulk–surface coupling. Deviations from the cylindrical shape would increase the bulk elastic contribution as well as raising the interfacial energy by changing the angle θ at the surface. For the approach to the stability analysis of fibers, refer to the works of Cheong and Rey (51, 52). The fibers become thinner with decreasing temperature (*SI Appendix*, Fig. S11), so the equation for $r_{f}$ suggests that the negative contributions to the interfacial free energy must increase with cooling. Assessing the effective interfacial tension experimentally requires measuring the Laplace pressure in the drop during the self-shaping process. For millimeter diameter drops, this particular task requires a lot of LC material and very sensitive pressure sensors due to the smallness of Laplace pressure. Laplace pressure is increased in tens-of-micrometer diameter droplets; however, this requires a very complex setup consisting of a very sensitive pressure pump able to read both positive and negative pressures differentials in LC material, connected to the droplet interior. Moreover, the measurement would have to be performed without disturbing the bulk director field, which in a N LCs, influences the surface through elastic interactions. In view of the complexity of the required experimental technique, these measurements are left for future experiments.

In all the experiments described here, the decreasing interfacial energy while cooling is presumably due to the combined effect of LC order and both surfactants together at the interface and attributed to the unique nature of LCs (41, 53). A pictorial representation of the excessive order at the interface is shown in *SI Appendix*, Fig. S12. A similar argument was proposed in ref. 41 to explain the growth of the cholesteric fibers and the division of droplets at the cholesteric–SmA transition. In all the experiments, self-shaping of a droplet to fibers (N and smectic) is observed only when two different surfactants were used: one in the dispersed phase and the other in the continuous phase. If only a single surfactant is used (e.g., either monoolein in 8CB droplets suspended in pure water or pure 8CB droplets suspended in a water–CTAB solution), no shape transformation takes place. This scenario further strengthens the previous assumption of the excessive surface order layer organized by the synergy of two surfactants. Thus, LC elasticity and the conservation of the total volume accommodate a larger surface area by reshaping the droplet. Therefore, the interfacial energy’s dependence on temperature is due to the increased order parameter at the surface at lower temperatures, as shown schematically in *SI Appendix*, Fig. S12. It is worth mentioning that a somewhat similar effect is responsible for the spontaneous shape transformation of liquid alkane droplets in a CTAB water solution (6). It was demonstrated that the interfacial region of alkane droplets freezes at some temperature, creating a crystalline monolayer of a mixture of an alkane and CTAB molecules, which decreases the interfacial energy.

In summary, the shape transformations described here show the versatility gained by the manipulation of the interfacial free energy, achieved by a combination of a surfactant in the continuous phase and a cosurfactant in the dispersed phase. The surface-minimizing tendency of the interfacial tension must be overcome to form nonspherical shapes, while preventing direct molecular dissolution in the aqueous host. The utilization of the bulk elasticity of an anisotropic liquid to counterbalance the surface growth is essential to achieve stable, nonspherical shapes, which can then be tuned and reconfigured in real time via the temperature-controlled interfacial free energy. The tunable monodisperse emulsions and fibers of LCs reported here are controlled using the cooling rate rather than microfluidics, which is a significant advantage that could simplify and scale up the experimental procedures. The self-shaped fibers and vesicle structures can be used with the help of polymerization as a platform to realize optical waveguides, resonators, rigid colloidal particles, elastomeric actuators, textile-like fibrous networks, and microswimmers. If the LC material is made active, the coupling of shape control to self-propulsion could lead to more complex, life-mimicking, dynamical shape transformations. The interplay between the interfacial free energy and the bulk elastic free energy in our LC vesicles can be a model system to study the active control of membrane lipids and the dynamics of the cytoskeleton, seen in biological systems. Finally, it is worth noting that we only explored the simplest of the LC phases; other LC materials (e.g., chromonics, cholesterics, blue phases, and bent-core materials), that have very different elastic responses and different symmetries, could lead to new realms of self-shaping structures to explore.

## Methods

### Materials and Sample Preparation.

We used the following commercially available materials: an ionic surfactant CTAB (hexadecyltrimethylammonium bromide), a nonionic surfactant monoolein (1-Oleoyl-rac-glycerol, Sigma Aldrich), and an LC compound (4-Octyl-4′-cyanobiphenyl, Cr 21.1 °C SmA 33 °C N 40 °C I, Synthon). The molecular structures are shown in *SI Appendix*, Fig. S1. The nonionic surfactant was used as a cosurfactant to increase the coverage of amphiphilic molecules at the 8CB–water interfaces. In all our experiments, we doped 8CB with 2 wt% monoolein before mixing 8CB with aqueous CTAB solutions to produce the emulsions. We also took care of the unwanted micellar solubilization of 8CB by working at CTAB concentrations that are well below the CMC (0.92 mM at 25 °C). Smectic vesicles in Fig. 4 are produced by using smectic LCs: 8OPhPy8 (5-Octyl-2-(4-octyloxyphenyl) pyrimidine, Cr 28.5 °C SmC 55.5 °C SmA 62 °C N 68 °C I) and C7 (4-(2S,3S)-(2-Chloro–3-methylpentanoyloxy)-4′-heptyloxybiphenyl, Cr 55.0 (SmG* 44.0, SmC* 55.0) SmA* 62.0 I) by doping with 1 weight percent (wt%) and 2 wt% monoolein, respectively. In *SI Appendix*, we present additional experiments using the N compounds 5CB, E7, and CCN37 for demonstrating the self-shaping phenomena shown in *SI Appendix*, Fig. S8. The compound 8OPhPy8 forming the SmC phase was used to grow helical fibers from a single droplet, as shown in Fig. 4. In a large number of experiments, 19 different LCs were used in combination with monoolein and CTAB to demonstrate the self-shaping of LC droplets into various shapes, such as straight and helical fibers, shells, and stomatocyte- and star-shape vesicles. This is a strong indication of the universality of our approach with two surfactants.

Though the self-shaping process is possible with a simple direct contact of monoolein-doped 8CB with an aqueous CTAB solution, we have used the following method in our experiments. Firstly, we prepared a small amount of stock emulsion consisting of polydisperse LC droplets in a 0.01 mM aqueous CTAB solution. Then, a small drop of the emulsion was placed between two glass plates, and 0.5 mM aqueous CTAB solution filled the cell through capillary action. We chose a combination of surfactant concentrations such that there was no possibility of micelles or any other surfactant-related, self-aggregated structures such as vesicles and lamellar phases in the aqueous medium. The presence of micelles leads to the unwanted dissolution of the LC in the hydrophobic core of micelles. It should be noted that the observed self-shaping structures still form in highly concentrated micellar phases, but the system becomes extremely dynamic and hard to follow.

### Cell Condition and Optical Microscopy.

The cell gap was adjusted with polymer spacers and held at 40 °C before the microscopic observation. The cell was sealed with 2 min epoxy. A Linkam THMSG600 heating stage was used to control the temperature of the cell. In this way, we followed the complete self-shaping process. We used a color camera (Canon, EOS 800D) mounted on the polarized optical microscope (Nikon LV 100POL) for observing the self-shaping process.

## Supplementary Material

Supplementary File

Supplementary File

Supplementary File

Supplementary File

Supplementary File

## Data Availability

All study data are included in the article and/or supporting information.
