# Supplementary material for: Self-shaping liquid crystal droplets by balancing bulk elasticity and interfacial tension
Source: Proc Natl Acad Sci U S A. 2021 Mar 31;118(14):e2011174118. doi: 10.1073/pnas.2011174118 (PMC8040587; doi:10.1073/pnas.2011174118)
Supplement: Supplementary File [file pnas.2011174118.sapp.pdf]

PNAS
www.pnas.org

Supplementary information for

Self-shaping of liquid crystal droplets by balancing bulk elasticity and interfacial tension

Karthik Peddireddy [1,2], Simon Čopar[3], Khoa V. Le [4], Igor Muševič[3,5], Christian Bahr[1], Venkata. S. R. Jampani [1,5,6]*

[1]Dynamics of Complex Fluids, Max Planck Institute for Dynamics and Self-Organization, Am Fassberg 17, 37077 Göttingen, Germany.

[2]Department of Physics and Biophysics, University of San Diego, 5998 Alcala Park, San Diego, CA, 92110, United States.

[3]Faculty of Mathematics and Physics, University of Ljubljana, Jadranska cesta, 1000 Ljubljana, Slovenia.

[4]Department of Chemistry, Faculty of Science, Tokyo University of Science, 1-3 Kagurazaka, Shinjuku-ku, Tokyo 162-8601, Japan.

[5]Condensed Matter Physics Department, Jožef Stefan Institute, Jamova 39, 1000 Ljubljana, Slovenia.

[6]Physics and Materials research unit, University of Luxembourg, Avenue de la faiencerie, 162a, L-1511, Luxembourg.

* Venkata. S. R. Jampani

Email: softmatter@vsrjampani.com

This PDF file includes:

Figures S1 to S12
Tables S1
Supplementary text
Legends for Movies: S1 to S4
SI References

Other supplementary materials for this manuscript include the following:

Movies S1 to S4

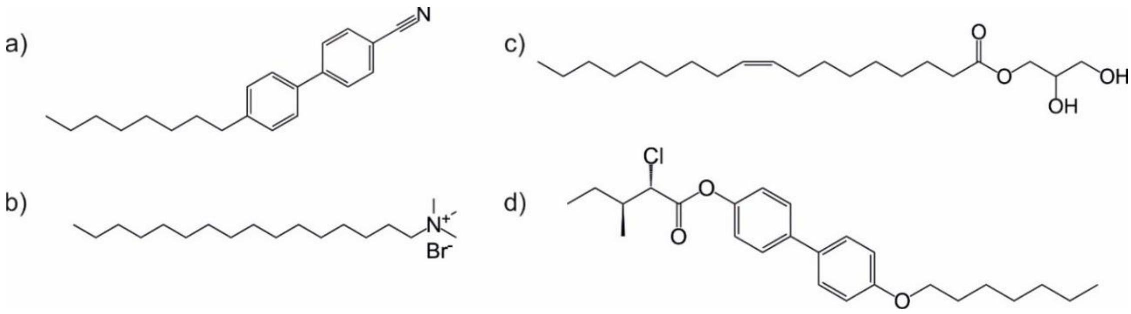

Fig. S1. Molecular structures of main materials used in this study. (a) 8CB, (b) CTAB, (c) Mono-olein, and (d) C7 liquid crystal.

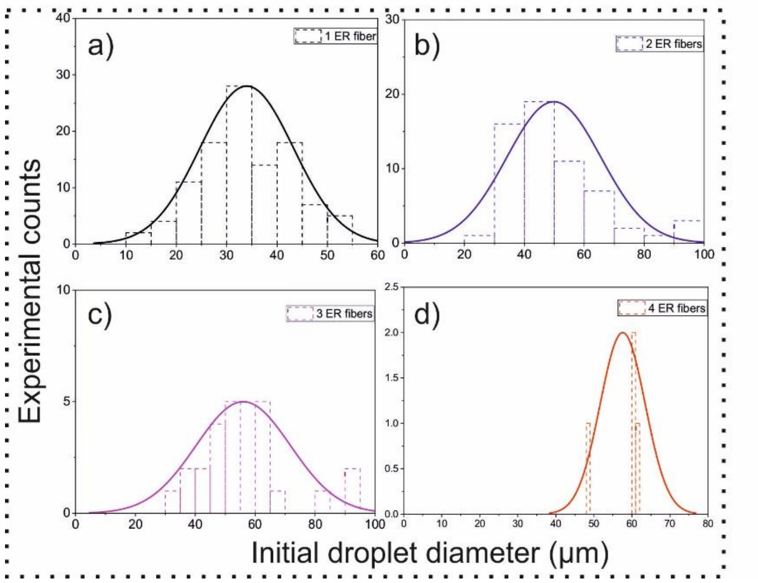

Fig. S2. The experimental investigation of ER fiber formation probability. (a-d) The experimental observations of one to four ER fibers at different initial radial droplet diameters at fixed surfactant concentration. The cooling rate of 2°C/min was used in all experiments. Note that 1ER fiber formation is more likely than 4ER fibers under any given condition. All the measurements were carried out with 2wt% mono-olein doped 8CB dispersed in 0.5mM CTAB solution between 39°C – 36°C.

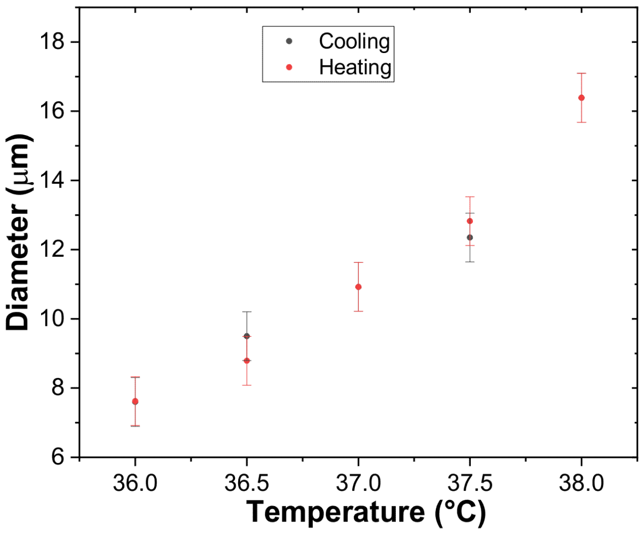

Fig. S3. A fully reversible temperature dependent shape transformation. A self-shaping droplet shows a fully reversible change in the diameter of the fiber with the cooling and heating cycle. See also Movie S1.

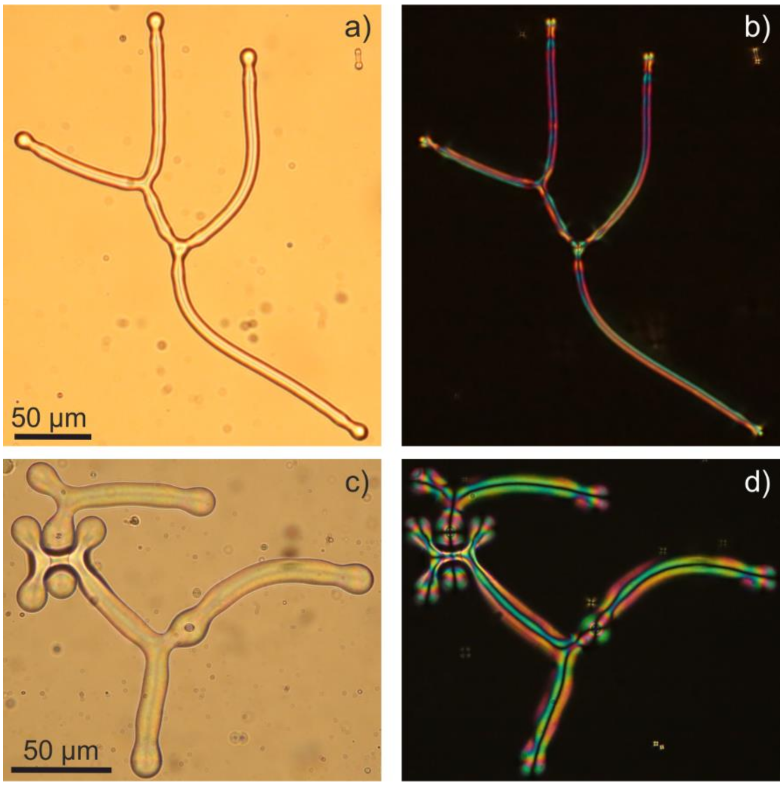

Fig. S4. Branching out of an ER fiber during the shape transformation from a radial droplet. Images with and without cross polarizer of two different complex ER structural patterns produced due to the further splitting of an ER fiber. The radial droplet is centered on all images. See also Movie S2.

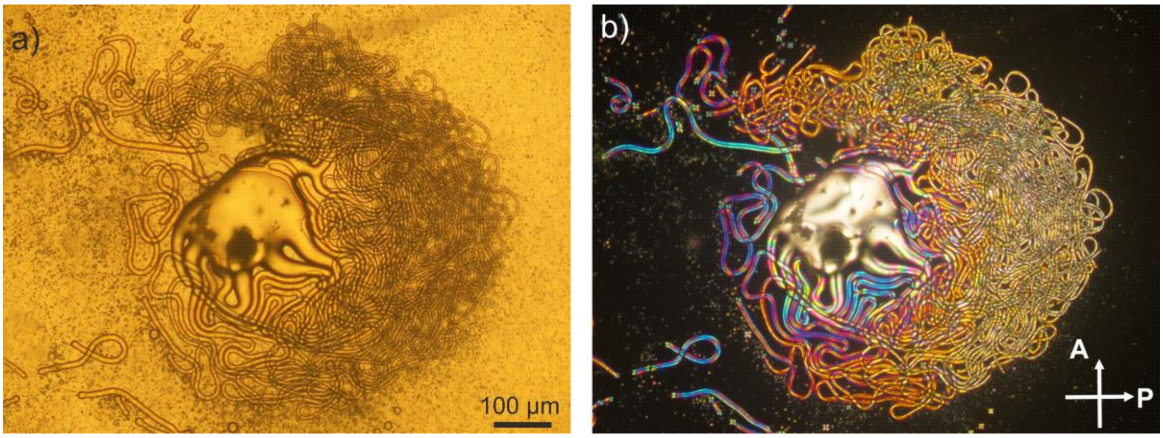

Fig. S5. A bundle of uniform ER fibers grown from an initially large nematic droplet. (a-b) The snapshots are taken at 36°C without and with crossed polarizers, respectively.

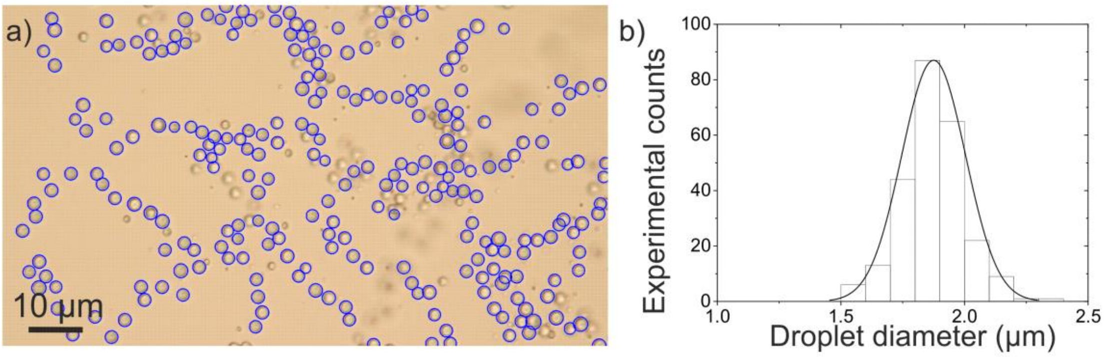

Fig. S6. The increase of elastic constant ratio during the $T_{N\text{-}SmA}$ transition transforms all ER fibers into highly monodisperse SmA droplets. (a) Droplets in the picture are produced by multiple fibers cooled below the N-SmA phase transition temperature (Movie S3). Out-of-focus droplets are omitted from the analysis. Droplet diameters (blue circles) are identified using a MATLAB function (imfindcircles). (b) A histogram of the droplet diameters shows minimal polydispersity. Please note that measured polydispersity is an overestimate as droplets are not in the same focal plane.

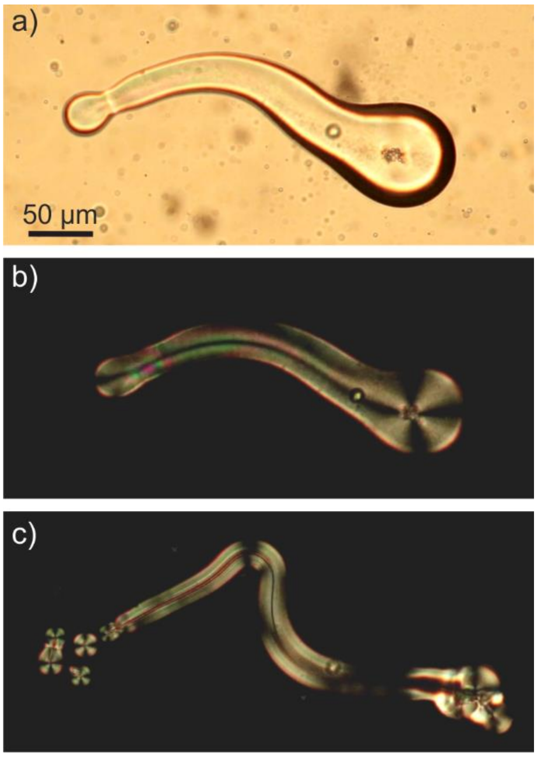

Fig. S7. A reversible transformation between nematic and smectic fibers is possible in highly viscous aqueous environments. (a-b) White light and polarizing optical images of a nematic fiber in an aqueous PEO-CTAB solution at 34 °C. The concentrations of polyethylene glycol (PEO) and CTAB in water are 5 wt% and 0.5 mM, respectively. The viscosity of water increases exponentially with increasing PEO concentration. (c) The system is quenched to the smectic phase (32 °C) at 20 °C/min. Note that structures are stable, no significant change has been detected for one-hour duration at the same temperature.

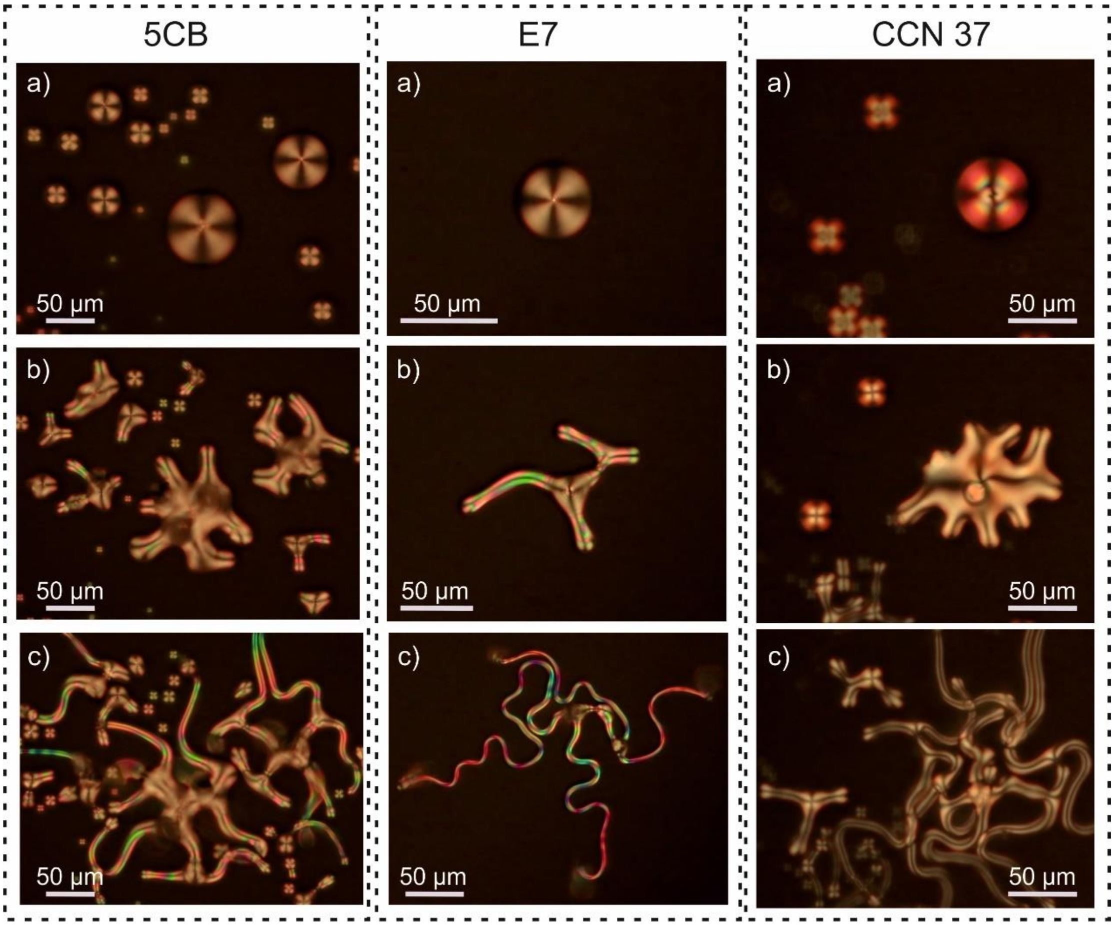

Fig. S8. Demonstration of self-shaping phenomenon in various LC compounds. (a-c) Standard liquid crystal; 4-Cyano-4'-pentylbiphenyl (5CB, Cr — 18°C — N — 35°C — I), E7 (eutectic LC mixture of cyanobiphenyls, Cr — -58°C — N — +60° — I) and a low birefringent LC material - CCN37 (4'-butyl-4-heptylbicyclohexyl-4-carbonitrile, Cr — 22.5°C — Sm A (18°C) — N — 54.1°C — I). The mono-olein and CTAB concentrations were used for 5CB - 0.6wt%, 0.7mM, E7 – 0.75wt%, 0.65mM, and CCN37 – 2wt%, 0.5mM respectively. The self-shaping temperatures 20°C, 28°C and 35°C for 5CB, E7, and CCN37, respectively. Note that our method shows self-shaping in all LC materials we have tried so far without fail.

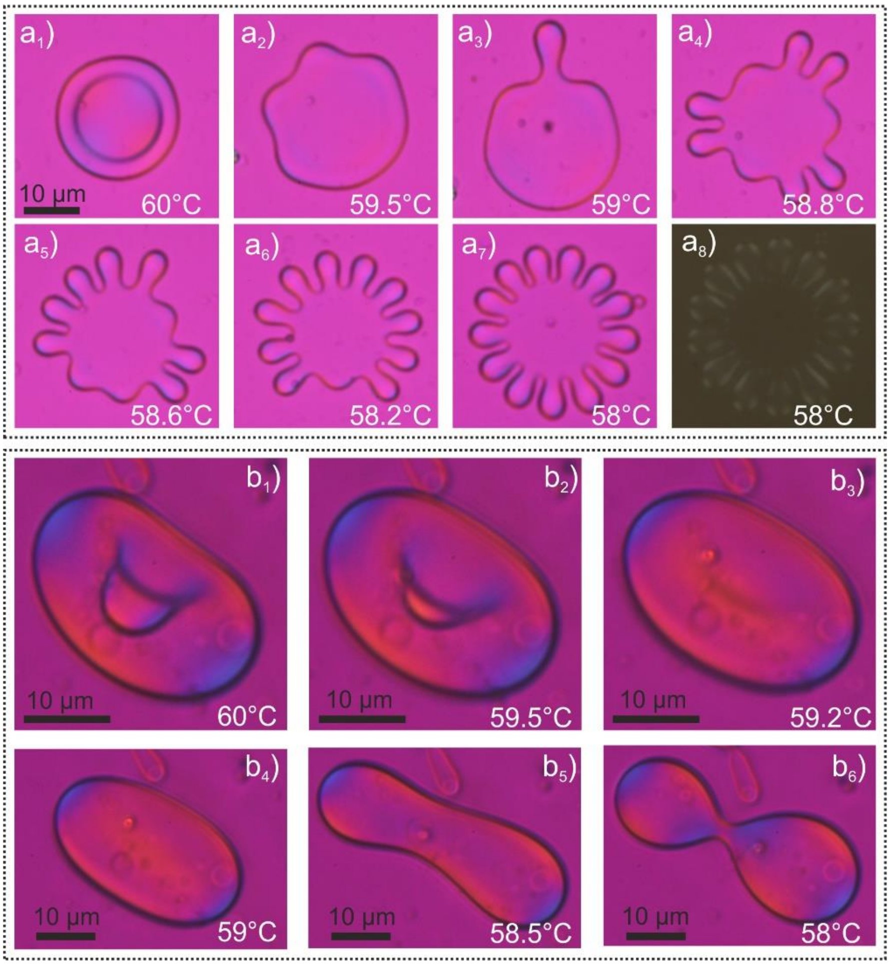

Fig. S9. Evolution of life-like characteristics in a SmA* LC (C7) vesicles through changes in volume to surface area ratio. (Panel a) Evolution of a starfish shape from an oblate structure. The number of budding events increases with a decrease in temperature. (Panel b) Another typical vesical shape transformation sequence (stomatocyte – discocyte – elliptocyte – dumbbell; all mimicking the shapes of human red blood cells) that is analogously observed in biomembranes. Gradual cooling produces the sequence. Except for panel a_8, which is taken between crossed polarizers, all other images were taken with an additional first order waveplate (530 nm) inserted between the polarizers. Note that experiments were carried out in the SmA* LC phase with the CTAB concentration in the aqueous phase is 1.5 mM, which is slightly above the CMC. Temperature is the only parameter that is varied from 60°C to 58°C to obtain all the observed fully reversible vesicle shapes.

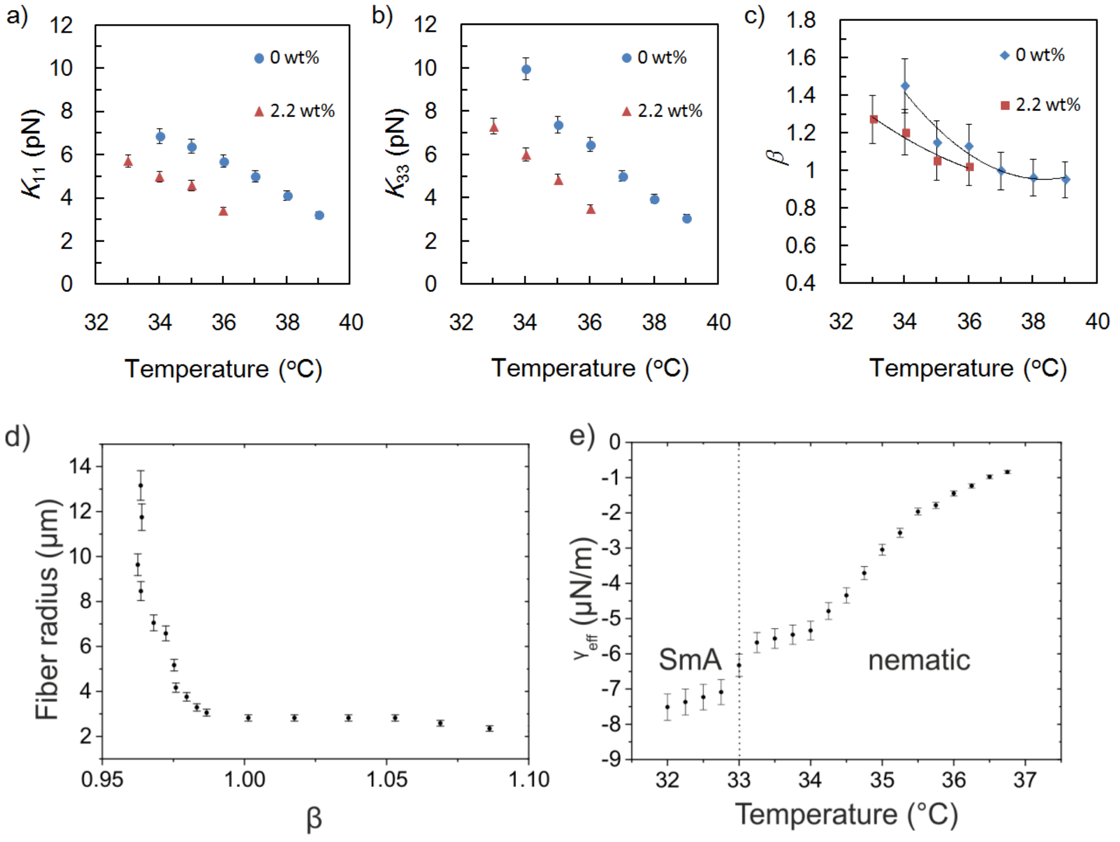

Fig. S10. Variation of elastic constants and their ratio as a function of temperature of pure 8CB and that mixed with Monoolein. (a) Splay elastic constants K_{11}. (b) Bend elastic constants K_{33}. (c) Ratios of bend to splay elastic constants β (=K_{33}/K_{11}). 0 wt% and 2.2 wt% represent the neat 8CB, and that mixed with 2.2 wt% Mono-olein, respectively. Lines in (c) are guides to the eye. We observe that with mono-olein added, the temperature effect on the elastic constant ratio is somewhat lessened, but still present. (d) The decreasing trend of the fiber radius to the elastic constant ratio. (e) Effective interfacial tension calculated from the ER fiber diameter, measured while cooling to SmA transition.

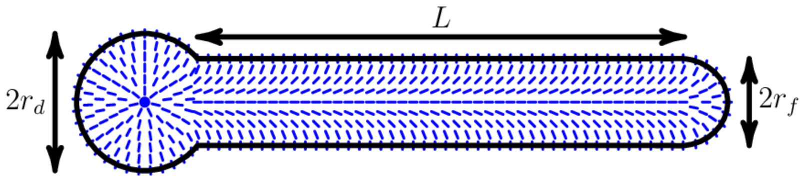

Fig. S11. Schematic representation of variables for fiber shape calculation. The droplet part containing a defect has a radial spherical profile and larger radius than the fiber, which contains an escaped radial profile. The blue rods represent the director field.

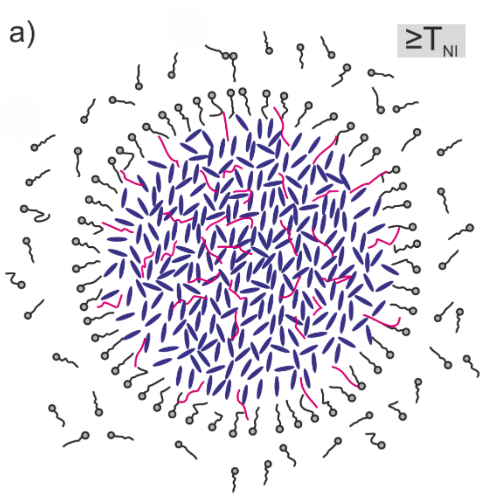

Fig. S12. Schematic representation of LC ordering at the surfactant covered surface. (a) The surfactant coverage at the interface above T_{NI}. (b) The surfactant promotes homeotropic alignment below T_{NI}. The magenta color represents the co-surfactant Mono-olein, which is bound with CTAB surfactant in water at the LC/water interface.

Table S1: A list of different LC materials tested so far with the combination of surfactants. As the self-shaping phenomena depend on the droplet diameter, the given temperature window is suitable for droplets of diameter starting from 15μm and above. Note that additional LC compounds: EBBA, 6CB, and 7CB were tested; however, details are not yet added to the list due to limited experimental data available.

LC compound	Concentrations of<br><br>Mono-olein (wt%) : CTAB (mM)	Self-shaping temperature window
5CB (Cr 18°C N 35°C I)	0.6 : 0.7	20°C – 16°C
8CB (Cr 21.1°C SmA 33°C N 40.5°C I)	2 : 0.5	37.5°C – 32°C
9CB (Cr 42°C SmA 48°C N 49.5°C I)	2 : 0.5	47°C – 42°C
10CB (Cr 44°C SmA 54.5°C I)	2 : 0.55	51°C–48°C
12CB (Cr 48°C SmA 58.5°C I)	2 : 1	53°C – 49°C
MBBA (Cr 22°C N 48°C I)	0.4 : 0.6	28°C – 23°C
E7 (Cr -58°C N +60° I)	0.75 : 0.65	28°C – 23°C
CCN37 (Cr 22.5°C Sm A (18°C) N 54.1°C I)	2 : 0.5	35°C – 30°C
CCN47 (Cr 28°C Sm A 33°C N 61.1°C I)	2 : 0.6	58°C – 54°C
CCN57 (Cr 19°C Sm B (17°C) Sm A 38°C N 67.5°C I)	2 : 0.6	62°C – 59°C
8OPhPy8 (Cr 28.5°C Sm C 55.5°C Sm A 62°C N 68°C I)	1 : 1.5	40°C – 34°C
C7 (Cr 55°C (SmG* 44°C SmC* 55°C) SmA* 62°C I)	2 : 1.5	60°C – 58°C
8-O-4 (Cr 55°C SmC 66°C N 89°C I)	1 : 1	64°C – 60°C
L-α-phosphatidylcholine	0 : 80	25°C – 23°C
8CB + CB15	2 : 0.7	26°C – 23°C
E7 + R811	2 : 0.75	45°C – 30°C
CCN55 + S811	2 : 0.65	46°C – 23°C

Supplementary Information Text

In order to investigate the self-shaping transformation, we have carried out many experiments at different initial diameters of the radial droplets in the same concentration of surfactant solution (0.5 mM CTAB, cooling rate 2°C/min). Figure S2 shows the statistics of these measurements (summarized in Fig. 1e). The probability of transforming a radial nematic droplet into a single escaped radial (ER) fiber is very high within the 0 – 30 µm droplet diameter range, and the possibility of getting two and three ER fibers is maximum when the initial droplet size is $\geq$ 60 µm. The total number of ER fibers grown out of the initial radial droplet increases with the increase of the radial droplet diameter. We propose that the limitation to four fibers arranging around the central droplet arises geometrically from the limited surface area on the droplet when it shrinks to its equilibrium size. Note that the probability of forming more than one fiber increases with an increase in the cooling rate.

Figure S3 plots the fiber diameter measurements for the complete cycle of a single radial droplet to one ER fiber formation and back, shown in Figure 1 in the main text and Movie S1. The initial diameter of the droplet is 17 µm at 38 °C, and the ER fiber diameter is measured to be 7.6 µm after cooling down to 36 °C at a rate of 1 °C/min. We have assigned the error of 3 pixels to all data points, which corresponds to 700 nm in the measurement.

Figs. S4 and S5 compare the structures grown from a small and an oversized initial droplet. In both cases, all the fibers grow until they equilibrate to the same diameter. Fig. S4 shows two cases with the central droplet at equilibrium diameter, and fibers further branched into multiple arms. Fig. S5 shows a droplet so large that it acts like an irregularly shaped reservoir of material, from which a dense bundle of fibers grows. The droplet itself is not at equilibrium with the interfacial energy and continue self-shaping into many more fibers of the same thickness.

Instability of an ER fiber into monodisperse droplets occurs approximately when the elastic constant ratio ($\beta = K_{33}/K_{11}$) exceeds 1, but the change in diameter is not instantaneous, so the fiber diameter at the time of breakup and the diameter of the created droplets depends on the cooling rate (Fig. 3 and Fig. S6). As an example, Movie S3 shows the whole process from radial droplets to monodisperse emulsions.

We have also studied the stabilization of LC fibers against jet instabilities induced by the phase transitions. Stabilization is achieved using polymer additives to the aqueous surfactant medium. We have used polyethylene oxide (PEO, average molecular weight: 200k) as an additive to the aqueous phase in order to overcome jet instability. The role of increased aqueous phase viscosity slows down the growth process drastically and improves the stability of the structure due to the viscous shear stress and partially due to the slowed diffusion of CTAB molecules to the interface. Thus, the nematic fiber does not fully break up during the phase transition, only producing a few droplets before stabilizing (Fig. S7). The same phenomenon is also observed with dextran (average molecular weight: 450k - 650k) as a thickening agent; therefore, it is not specific to any polymer additive. The N-SmA transition requires re-orientation of molecules from nematic ER state to radial SmA structures, as it is shown below in Fig. S7. Such a state, therefore, undergoes an inevitable volume change during the radial alignment of SmA layers. We still have observed the formation of a few Sm-A droplets at the tip of the fiber –away from the hedgehog defect- during the N-SmA transition, attributed to an abrupt change in the elastic constant ratio during the phase transition. Further study is necessary to understand this stabilization method in full.

In another experiment, we have used C7 –a smectic LC with no nematic phase in its phase sequence- doped with 2 wt% co-surfactant for realizing the curved structures i.e., thermotropic

vesicles (Figs. 4 in the main text, and Fig. S9). Vesicles readily form if CTAB concentration slightly exceeds critical micelle concentration (CMC). Vesicles are very stable structures that show no change in their shapes as long as the experimental conditions are maintained. However, as with nematic filaments, thermotropic vesicle shapes can be reversibly controlled by varying the temperature. As seen in Fig. S9, Vesicle shape dynamics seems identical to the dynamics observed in biomembranes (lyotropic structures) despite the differences in the composition and the methods of production.

Several models are developed to explain biomembrane dynamics. However, investigating effects such as phase transitions on vesicle shapes is still an active area of research. Owing to the simplicity of thermotropic vesicle composition and with already existing well-developed theories for thermotropic liquid crystals, we believe this new system should be able to provide more insights into vesicle shape dynamics.

Elastic constant measurements: The elastic constants are measured as follows. First, the splay elastic constant K_{11} is measured from the threshold voltage Vth of Fréedericksz transition in a homogeneous cell using the following formula.

$$V_{\mathrm{th}} = \pi \sqrt{\frac{K_{11}}{\varepsilon_0 \Delta\varepsilon}}$$

Here ε_0 is the vacuum permittivity, and $\Delta\varepsilon$ is the dielectric anisotropy ($\varepsilon_{\parallel}$-$\varepsilon_{\perp}$) of the sample.

Next, the bend elastic constant K_{33} is measured using a capacitance method. When a voltage is applied across the LC cell, the capacitance C starts to change from a threshold voltage Vth. By a numerical curve fitting to the C-V data, the quantity $\frac{K_{33}}{K_{11}} - 1$ is obtained, and K_{33} can be extracted if K_{11} is known.

Our results are shown in Fig. S10. For the neat 8CB sample, the values are in good agreement with the reported data. In homogeneous mono-olein doped samples, there is a significant downshift of the elastic constants due to reduced order induced by the mono-olein in bulk as an impurity. Note that the mono-olein is likely inhomogeneous in the LC emulsion experiments and expected to localize to the interface, thereby actively reducing the interfacial energy. Thus, assuming the mono-olein concentration is low in the bulk of the fibers, we have used neat 8CB elastic constant values from the measurements for plotting the fiber radius as a function of $\beta = \frac{K_{33}}{K_{11}}$ (Fig. S10d). While β does vary with temperature, its change is not significant enough to account for the change in radius. Rather the interfacial free energy must also change (see Fig. S10e). There is a downshift of 1°C in $T_{I\text{-}N}$ transition in emulsions (Movie S4). Significantly, the fiber radius is decreasing monotonically until the β value reaches 1. If β is more than unity, the bend elastic constant is dominating, and the fiber growth is stopped. Eventually, during the transition, the fiber destabilizes and breaks into monodisperse droplets.

Phenomenological equilibrium model: Based on the experimental results, we constructed a simple phenomenological model of a droplet-fiber system. Combining bulk elasticity, surface free energy connected to the effective interfacial tension (γ_{eff})—a combination of isotropic interfacial tension and the entropic and enthalpic contributions to the free energy of the surfactant-cosurfactant layer interacting with the liquid crystals at the interface, which is anisotropic due to the surface anchoring—and Lagrange multiplier (pressure p) that enforces the constant volume constraint during minimization, we obtained the Gibbs free energy (G),

composed of the droplet (first parentheses) and fiber (second parentheses) contributions (for geometric parameters, see (Fig. S10)):

$$G = \left(8\pi K_{11} r_d + 4\pi r_d^2 \gamma + \frac{4}{3}\pi r_d^3 p\right) + \left(\Omega \pi K_{11} L + 2\pi r_f \gamma L + \pi r_f^2 L\, p\right).$$

In a droplet with a radial director structure, the bulk energy is solely due to splay deformation and scales linearly with droplet radius r_D: $F_{d,Bulk} = 8\pi K_{11} r_d$. The fiber contributions are based on escaped radial structure, for which the energy was derived for general splay and bend elastic constants by Cladis and Kleman (Ref S1),

$$F_{f,Bulk} = \Omega \pi K_{11} L, \qquad \Omega = 2 + \beta \frac{\arctan\sqrt{\beta - 1}}{\sqrt{\beta - 1}}, \qquad \beta = \frac{K_{33}}{K_{11}}.$$

The pre-factor Ω increases upon cooling to the smectic phase, as the bend elastic constant increases relatively to the splay elastic constant. In the above model, only the effects of splay and bend were considered. If finite surface anchoring, saddle-splay elasticity, or different director textures, such as twisted escaped structure or a singular core structure, are considered, the Ω factor assumes a more complicated form, but the contribution to the free energy is still proportional to a positive multiple of the fiber length, therefore the conclusions of this analysis remain unchanged. The birefringence signature of our experimental images confirms that the anchoring is strong enough that the effect of angle deviations from perpendicular can be ignored. Moreover, saddle-splay cannot reverse the sign of the surface-scaling energy term if K_{24} elastic constant conforms to Ericksen's consistency inequalities [S3, S4]. Except for the temperatures right before the N-SmA transition, all aforementioned effects are small, and the value of the pre-factor is reasonably close to the value obtained in approximation of equal elastic constants, $\Omega = 3$.

Hydrostatic equilibrium is achieved when pressure is equal in both parts of the contiguous internal volume (Fig. S11). Minimization of the free energy functional over fiber length L, both radii r_f, r_d, under a constant volume constraint, yields the equilibrium values for the fiber radius and the ratio of radii of the droplet and the fiber.

$$r_f = -\frac{\Omega K_{11}}{\gamma_{eff}}, \quad \frac{r_d}{r_f} = 1 + \sqrt{\frac{\Omega - 2}{\Omega}}$$

A similar argument can be made to break up into droplets at the N-SmA transition. The elastic part of the free energy of the escaped radial (ER) fiber starts out at $F_{ER} = 3\pi K_{11} L$. As the bend elastic constants diverge, the prefactor grows, and the ER structure is no longer energetically favorable. However, this frustration has two possible resolutions: breakup into droplets with pure splay distortion, or transforming a fiber with a singular core, and eventually concentric cylindrical layers in the smectic phase.

A simple energy estimate yields elastic energy of a singular core fiber with core radius $a \approx 2\ \mathrm{nm}$ as described in Cladis et al.,(Ref S2) (Reference. 38 in the main text), calculated at a typical droplet radius $R = 5\ \mu\mathrm{m}$ (Fig. 3g),

$$F_{cyl} = \ln\frac{R}{a}\, \pi\, K_{11} L \approx 8\, \pi\, K_{11} L,$$

and a train of droplets with the same radius and the same total volume,

$$F_{drops} = 6\,\pi\,K_{11}L.$$

Based on this estimate, the energy cost for a singular core fiber is higher than the equivalent cost of droplets, as well as having a smaller surface area, which for negative surface energy, is also in favor of multiple smaller droplets.

However, this estimate neglects the transition's dynamic nature, as the speed of the transition can influence which transition will occur. As shown in Fig. S7, increasing the surrounding medium's viscosity stabilizes the transition to a singular core fiber. Once the layer structure is assembled, break up into droplets would require costly rearrangement of layers through the formation of smectic focal planes, which stabilizes the resulting structure.

Microscopic mechanism: We suggest the interfacial free energy dependence on temperature is due to the increased order parameter at the surface at lower temperatures, as shown schematically in Fig. S12. In all our experiments shown in this report, the decreasing interfacial free energy while cooling was due to a combined effect of LC order and surfactants together at the interface and attributed to the unique nature of LCs. It is known that the co-surfactant (here, Mono-olein) promotes LC ordering at the LC water/interface. This scenario creates an excess order at the interface and continues ordering while cooling into more ordered LC phases. Therefore, the whole self-shaping process is due to the synergistic effect of different contributions to the interfacial free energy and the LC bulk elastic free energy, manifested in the form of controllable and fully reversible LC superstructures. We leave the specifics of the underlying microscopic mechanism that manifests as negative total interfacial free energy at the level of phenomenological macroscopic description as an open question that requires additional research.

Movie S1 (separate file). The movie shows the reversible nature of radial to ER fiber formation from a single droplet. The cooling and heating rate is 1 °C/min, and the radial droplet diameter is 17 µm. Movie S1 started at 36.5°C (3 ER fibers) and heated to 38°C (radial droplet). Starting at 27sec in the video, the radial droplet undergoes a full temperature cycle: 38°C – 36.5°C – 38°C at 1°C/min, which resembles the transformation of a radial droplet to a single ER fiber and back.

Movie S2 (separate file). The movie shows the complete transformation of several polydisperse droplets into ER fibers and eventually destabilize into monodisperse droplets during nematic to Smectic A transition. The horizontal width of the movie is 733 µm. The cooling rate is 3 °C/min.

Movie S3 (separate file). The transformation of an ER fiber into monodisperse droplets while cooling from 40 °C to 32 °C. The cooling rate is 2 °C/min.

Movie S4 (separate file). Temperature dependent tunable ER fiber diameter. The movie is sped up by 16×. If the set cooling rate is higher than 12°C/min, we started to see a delay in the LC response from the set temperature readings, which is an instrumental problem. It is to be noted that this time delay in fiber formation gets significant with the increase of the cooling rate. The horizontal width of the movie is 247 µm. Note that the vertical temperature gradient might also affect the initiation of the self-shaping process, and thus the fiber diameters might vary by the droplet location at different heights.

SI References

1. P. E. Cladis, M. Kléman, Non-singular disclinations of strength S = + 1 in nematics. *J. Phys.* **33**, 591–598 (1972).

2. P. E. Cladis, S. Torza, Growth of a smectic A from a bent nematic phase and the

smectic light valve. *J. Appl. Phys.* **46**, 584–599 (1975).

3. J. L. Ericksen, Inequalities in Liquid Crystal Theory, *Phys. Fluids* **9**, 1205 (1966).
4. J. V. Selinger, Interpretation of saddle-splay and the Oseen-Frank free energy in liquid crystals, *Liquid Crystals Reviews* **6**, 129 (2018).
